# Targeting HLA-F suppresses the proliferation of glioma cells via a reduction in hexokinase 2-dependent glycolysis

**DOI:** 10.7150/ijbs.56357

**Published:** 2021-03-25

**Authors:** Xin Chen, Na Sun, Rongqin Li, Xuejia Sang, Xueqin Li, Jie Zhao, Jing Han, Jing Yang, Takayuki Ikezoe

**Affiliations:** 1 Jiangsu Province Key Laboratory of Immunity and Metabolism, Xuzhou Medical University, Xuzhou, China.; 2 Department of Pathogenic Biology and Immunology, Xuzhou Medical University, Xuzhou, China.; 3 China University of Mining and Technology, Xuzhou, China.; 4 The Department of Hematology, Fukushima Medical University, Fukushima, Japan.

**Keywords:** HLA-F, glycolysis, hexokinase 2, glioma

## Abstract

HLA-F, a nonclassical HLA class I molecule, is required for regulating immune tolerance. In recent years, HLA-F has been found to play a role in a variety of cancers, including glioma (GM). Additionally, high expression of HLA-F predicts the poor overall survival of individuals with GM. However, the functions of HLA-F in GM remain to be further elucidated. In this study, we found that HLA-F expression was elevated in GM tissues. High levels of HLA-F resulted in a high cell proliferation index and predicted GM recurrence. Forced expression of HLA-F promoted the growth of murine C8-D1A cells transplanted in immunodeficient *Rag2^-/-^* mice. In contrast, silencing HLA-F inhibited cell growth *in vitro*. Furthermore, targeting HLA-F with an anti-HLA-F antibody suppressed the growth of C8-D1A cells stably expressing HLA-F transplanted in immunodeficient *Rag2^-/-^* mice. In further experiments, we found that forced expression of HLA-F contributed to the aerobic glycolysis phenotype in C8-D1A cells along with an increase in HK2 protein stabilization. Conversely, silencing HK2 by shRNA reduced HLA-F-mediated glycolysis and cell proliferation. Our data indicated that HLA-F promoted cell proliferation via HK2-dependent glycolysis. HLA-F could be a potential therapeutic target for the treatment of GM.

## Introduction

The prognosis of individuals with GM, especially glioblastoma (GBM), is generally poor. It has been reported that the median survival time is approximately 14 months, and only 3-5% of patients can survive up to 5 years after diagnosis 1, 2. Hotspot mutations in tumor suppressors and proteins involved in regulating cell signaling and growth have been found in GM. Additionally, recent studies have demonstrated that higher levels of PD-L1 3, CD95 ligand (CD95L), and TGF-β 4, 5 were observed in GM patients, which suppresses immune responses and helps GM cells escape immune surveillance.

Alterations in HLA class I (HLA-I) expression have been found in a variety of malignancies. Additionally, these alterations could have a decisive influence on the tumor immune response and metastatic ability 6, 7. HLA-F, a nonclassical HLA class I molecule (HLA-Ib), is structurally related to HLA-Ia molecules. However, in contrast to HLA-Ia molecules, HLA-F has a limited tissue distribution and is generally considered a tolerogenic molecule 8, 9. HLA-F physically interacts with killer cell immunoglobulin-like receptors (KIRs), including KIR3DL2, KIR2DS4, and KIR3DS1 10, and is involved in regulating human pregnancy, infection, and autoimmunity 8, 11, 12. Moreover, recent studies have demonstrated that HLA-F is constitutively expressed in various cancers, including non-small cell lung cancer, gastric cancer, and GM 9, 13, 14. Notably, HLA-F expression is negatively associated with overall survival 9, 13, indicating that HLA-F might play a role in tumorigenesis or tumor progression.

In addition to the induction of immunosuppression, GM progression is associated with metabolic remodeling, resulting in aerobic glycolysis as the primary source of energy and biosynthetic precursors for proliferation 15. The glycolytic enzymes hexokinases (HKs), including HK1, HK2, HK3 and HK4, catalyze the first committed step in glucose metabolism and influence the magnitude and direction of glucose flux within cells 16, 17. HK1 is predominantly expressed in most mammalian adult tissues, including the normal brain. HK2 is abundantly expressed in skeletal, adipose tissues and cardiac muscles but not in the normal brain 18. Recent studies have demonstrated that HK2 is constitutively expressed in GBMs and plays a vital role in proliferation and cell survival 19, 20. HK2 suppresses apoptosis through translocation to the outer mitochondrial membrane and interaction with the permeability transition pore 21-23. Several transcription and growth factors have been indicated to modulate the expression and activity of HK2 24, 25. A recent study found that B1.23.2, an HLA-B, C-specific monoclonal antibody, decreased the levels of key glycolytic enzymes, glutamine transporters and glutaminase enzymes, reducing energy production and melanoma cell motility 26. Nevertheless, whether HLA-F influences HK2 expression or activity remains unclear.

This study identified a crucial role for HLA-F in regulating the proliferation of GM cells via HK2-dependent glycolysis.

## Material and Methods

### Cells

The murine astrocyte cell line C8-D1A was commercially obtained from Fuheng (FH0837, FuHeng Cell Center, Shanghai, China). Human GM U373, HS683 and U138MG cell lines were commercially obtained from ATCC. SHG44, U251, T98G, TJ905 and A172 cells were commercially obtained from Bolida (Bolida, Xuzhou, Jiangsu, China). The cells were cultured in DMEM containing 10% heat-inactivated FBS (FB25015, Clark, Virginia, USA), 100 mg/L streptomycin and 100 U/mL penicillin at 37 °C in a 5% CO_2_ environment. All human GM cell lines have been authenticated using STR profiling within the last three years. In addition, all experiments were performed with mycoplasma-free cells.

### Plasmids, shRNA and Transfection

Myc-tagged human HK2 cDNA was synthesized and cloned into the pcDNA3.1 (+) vector at the NheI/HindIII sites. shRNAs targeting human HLA-F or murine HK2 were purchased from GeneChem (GeneChem, Shanghai, China) or Jima (Jima., Shanghai, China), respectively. Cells were transfected with either control shRNA or the indicated shRNA utilizing jetPRIME transfection reagent (Polyplus, France) according to the manufacturer's instructions. After 24 h, the cells were harvested and used for further experiments. Puromycin (2 mg/mL, VICMED, Xuzhou, Jiangsu, China) was added to the culture medium to select the transfected cells. The shRNA sequences were as follows: shHLA-F #1: CGCAGTATTGGGAGTGGACCACTCGAGTGGTCCACTCCCAATACTGCGTTTTTG, shHLA-F #2: CCGGAGAGGAATATGCAGAGGAGTTCTCGAGAACTCCTCTGCATATTCCTCTTTTTTG; shHK2 #1: GATCCGCGTAGATGCATAACAAGATTCAAGAGATCTTGTTATGCATCTCTACGCTTTTTTG, shHK2 #2: GATCCGGAGATGCGTAATGTGGAACTTTCAAGAGAAGTTCCACATTACGCATCTCCTTTTTG, and shHK2 #3: GATCCGCATATGATCGCCTGCTTATTTCAAGAGAATAAGCAGGCGATCATATGCTTTTTG.

### Lentivirus and infection

HLA-F lentivirus was obtained from GeneChem (GeneChem.). Cells were infected with the indicated lentivirus according to the manufacturer's instructions. After 24 h, 1 mL culture medium was added to the wells. 24h later, the infected cells were cultured in DMEM culture medium containing 10% FBS and 2 mg/mL puromycin.

### Colony formation assay

The colony formation assay was performed as previously described 27. Briefly, 200 infected cells were cultured in six-well plates at 37 °C for 7-10 days. Visible colonies were washed twice with PBS, fixed with 4% paraformaldehyde, and stained with crystal violet. Images of the colonies were taken, and the number of colonies was counted by ImageJ software.

### Generation of anti-HLA-F antibody

Commercial anti-HLA-F antibodies are mainly used to detect proteins but cannot be used as blocking antibodies. We generated a patented monoclonal antibody (mAb) to solve this issue. Briefly, an HLA-F cDNA expression vector, pCzn1(+)-HLA-F, was constructed by inserting the fragment carrying the human HLA-F cDNA between the NdeI and XbaI sites of the pCzn1(+) expression vector (Zoonbio, Nanjing, China). The pCzn1-HLA-F expression vector was transformed into ArcticExpress competent cells and incubated with isopropyl-1-thio-β-D-galactopyranoside (IPTG) (final concentration was 0.5 mM) at 37 °C for 4 h with shaking. Then, the fusion protein was purified by Ni-iminodiacetic acid (IDA) affinity chromatography. The purified protein was confirmed by CBB staining and Western blot analysis (data not shown).

Six- to eight-week-old female BALB/c mice were immunized with 50 μg purified HLA-F protein emulsified in the same volume of complete Freund's adjuvant by intraperitoneal injection 28. Three additional injections were administered to the mice at 2-week intervals with the same dose of antigen emulsified in incomplete Freund's adjuvant 28. At the end of the experiments, immunized mice were sacrificed, and single spleen cells were fused with SP2/0 myeloma cells utilizing polyethylene glycol 1450 (PEG1450, Sigma-Aldrich, USA) 28, 29. After which, the hybridoma cells were seeded into 96-well plates and followed by selection in hypoxanthine-aminopterin-thymidine (HAT) medium and hypoxanthine-thymidine (HT) medium. The cell culture of surviving clones were assayed by indirect ELISA for antibody reactivity and specificity 28. One hybridoma was subsequently cloned by repeated limiting dilution until a stable clone was obtained 29. Ascitic fluids were produced in pristane-induced BALB/c mice 28.

### Indirect enzyme-linked immunosorbent assay (ELISA)

Indirect ELISA was used to determine the immune reactivity of HLA-F and to screen positive hybridoma cells as described previously 28. Briefly, ELISA plates were plated with purified HLA-F protein in PBS (pH 7.4), coated at 4 °C overnight and. After which, the plates were incubated with 100 μL diluted antibodies at 37 °C for 1 h, and followed by incubation with horseradish peroxidase (HRP)-conjugated goat anti-mouse IgG (Proteintech Group, China) at a 1:5000 dilution in PBST at 37 °C for 1 h. After washing, 50 μL/well of TMB substrate (Amresco, Solon, Ohio, USA) was added into the wells, and the plates were incubated for 15 min at room temperature in the dark. OD450 values were obtained after stopping the reaction with 1 M HCl (100 μL/well).

### Ectopic tumor implantation and weight measurements

The mouse strains were maintained and housed under specific pathogen-free (SPF) conditions. Six-week-old female *Rag2^-/-^* mice (Shanghai Model Organisms Center Inc., Shanghai, China) were utilized for ectopic implantation. The mice's left and right flanks were shaved, and 1×10^6^ C8-D1A cells were injected subcutaneously (s.c.). For monoclonal antibody experiments, when a palpable mass formed, the mice were divided into two groups and treated with either the same volume of control diluent or 100 μg/mass anti-HLA-F mAb by intratumor injection. The palpable mass size was monitored every other day. The detectable mass size was measured three times a week using calipers. The palpable mass sizes were calculated using the formula a×b×c, where “a” is the length, “b” is the width, and “c” is the height in millimeters. 14 days later, the animals were euthanized, and the masses were isolated to determine the weight following careful dissection.

### 3-(4,5-Dimethylthiazol-2-yl)-2,5-diphenyltetrazolium bromide assays

The proliferation of cells was assessed by 3‐(4,5‐dimethylthiazol‐2‐yl)‐2,5‐diphenyltetrazolium bromide (MTT) assay. Briefly, cells were transfected with either control shRNA or shRNA against HLA-F. After 24 h, cell viability was measured by the MTT assay.

### RNA isolation and real-time reverse transcription-polymerase chain reaction (RT-PCR)

We measured the expression of *18S* or *Actb* for normalization as previously described 27. Real-time RT-PCR was performed using TB Green PCR Master Mix (TaKaRa, Japan). The primer sets for PCR are shown in Table 1. The PCR conditions for all the genes were as follows: initial activation at 95 °C for 30 s, followed by 40 cycles at 95 °C for 5 s, 60 °C for 20 s, and fluorescence determination at the melting temperature of the product for 15 s on a LightCycler 480 (Roche).

### Determination of mRNA half-life

To measure the half-life of endogenous *Hk2* mRNA, cells were cultured in the presence of either control diluent or actinomycin D at a final concentration of 2 µg/mL at the indicated time points, the total RNA was extracted and subjected to real-time RT-PCR. mRNA levels were normalized to *18S* levels and plotted as a percentage of the value at time 0 (set at 100%).

### Immunoblotting

Immunoblotting was performed as described previously 30, 31. Anti-HLA-F (14670-1-AP), -HK1 (19662-1-AP), -HK2 (22029-1-AP), -PKM1 (15821-1-AP), -PFKM (55028-1-AP), -Myc (16286-1-AP), and -β-actin (66009-1-Ig) antibodies were purchased from Proteintech.

### Immunoprecipitation

293T cells were cotransfected with the indicated plasmid DNA. After 24 h, lysates were prepared in immunoprecipitation lysis buffer (20 mmol/L Tris-Cl, pH 8.0, 10 mmol/L NaCl, 1 mmol/L EDTA, 0.1% NP-40) containing a protease inhibitor cocktail (Sigma-Aldrich). One microgram of cell extract was precleared with 50 µL of protein A/G-agarose (Thermo Fisher) at 4 °C for 15 min. After centrifugation, the supernatant was harvested and incubated with the corresponding antibodies with gentle shaking at 4 °C for overnight, followed by the addition of 20 µL of protein A/G-agarose for another 1 h. The beads were washed, resuspended in 30 µL of 1×loading buffer and boiled for 3 min, followed by Western blot detection.

### Protein half-life assay

Cells were treated with cycloheximide (CHX, final concentration: 100 μg/mL) and harvested using sodium dodecyl sulfate (SDS) lysis buffer at the indicated time points. The levels of HK2 and β-actin were analyzed by Western blotting. HK2 bands were quantified after normalization to β-actin, and the data were plotted as the relative amount of protein remaining compared to the treatment at time zero. Bands were compared quantitatively using ImageJ software.

### Immunofluorescence staining and quantification

A tissue microarray of the GM cohort consisting of 180 cases (OUTDO IVD, Shanghai, China) was used in the study. Staining was performed with an antibody against HLA-F together with immunofluorescent markers (Opal, PerkinElmer) and digital images acquired by a multispectral imaging system (PerkinElmer). Quantification of positively stained cells was verified with manual counting.

### Seahorse analyzer

The extracellular acidification rate (ECAR) was measured with an XF 24 extracellular flux analyzer (Seahorse Bioscience). Briefly, 5×10^4^ cells were seeded in each well of Seahorse XF 24 plates with 250 μL of DMEM and incubated overnight. ECAR measurements were normalized to the cell number. In ECAR tests, the cells were plated in XF Seahorse media with 2 mM glutamine using the following concentrations of injected compounds, as indicated in the text: 1 µM oligomycin, 500 mM 2-DG, and 100 mM glucose. For the oxygen consumption rate (OCR), the cells were plated in XF Seahorse media with 10 mM glucose, 2 mM glutamine, and 1 mM sodium pyruvate in the mitochondrial stress test using the following concentrations of injected compounds: 1 µM oligomycin, 1 µM Carbonyl cyanide 4-(trifluoromethoxy)phenylhydrazone (FCCP), and 100 nM rotenone + 0.5 µM antimycin A (Seahorse Bioscience), as indicated.

### G6PDH activity

G6PDH activity was examined using G6PDH Activity Assay Kit with WST-8 (Beyotime, Shanghai, China) according to the manufacturer's instructions.

### The expression of HLA-F and survival analysis using the GEPIA web tool

The online database Gene Expression Profiling Interactive Analysis (GEPIA2, http://gepia2.cancer-pku.cn/#index) was used to analyze the expression of HLA-F and the overall survival (OS) of individuals with various types of cancers.

### TCGA data re‐analysis

The gene expression profiles and clinicopathological information of a large cohort of low-grade glioma (LGG) patients (509 cases) in The Cancer Genome Atlas (TCGA) were obtained from the Genomic Data Commons Data Portal (GDC) website (https://portal.gdc.cancer.gov/). Data collection and application were performed in accordance with TCGA publication guidelines and data access policy without the need for additional approval from the local ethics committee. The downloaded RNA expression data were scored by the ESTIMATE algorithm for stromal scores, and LGG cases were classified into high and low groups based on the median score. Data analysis was performed using the “limma” package (version 3.8) in R (version 3.6.0).

### Statistical analysis

To compare two groups, Student's t-test was utilized. One-way ANOVA followed by multiple comparisons test was used to compare the differences between more than two groups. Kaplan-Meier univariate survival analysis was performed by classification as 'low' or 'high' based on the median densities of cells, and *P* values are reported using the log-rank (Mantel-Cox) test with HR and 95% CI as described in a previous study 32. The association between HLA-F expression and patient clinicopathological parameters was evaluated by chi square (χ^2^) analysis. Both tests were performed using GraphPad Prism V5 (GraphPad Software).

## Results

### Constitutive expression of HLA-F predicted poor outcomes in GM patients

**Figure 1:**
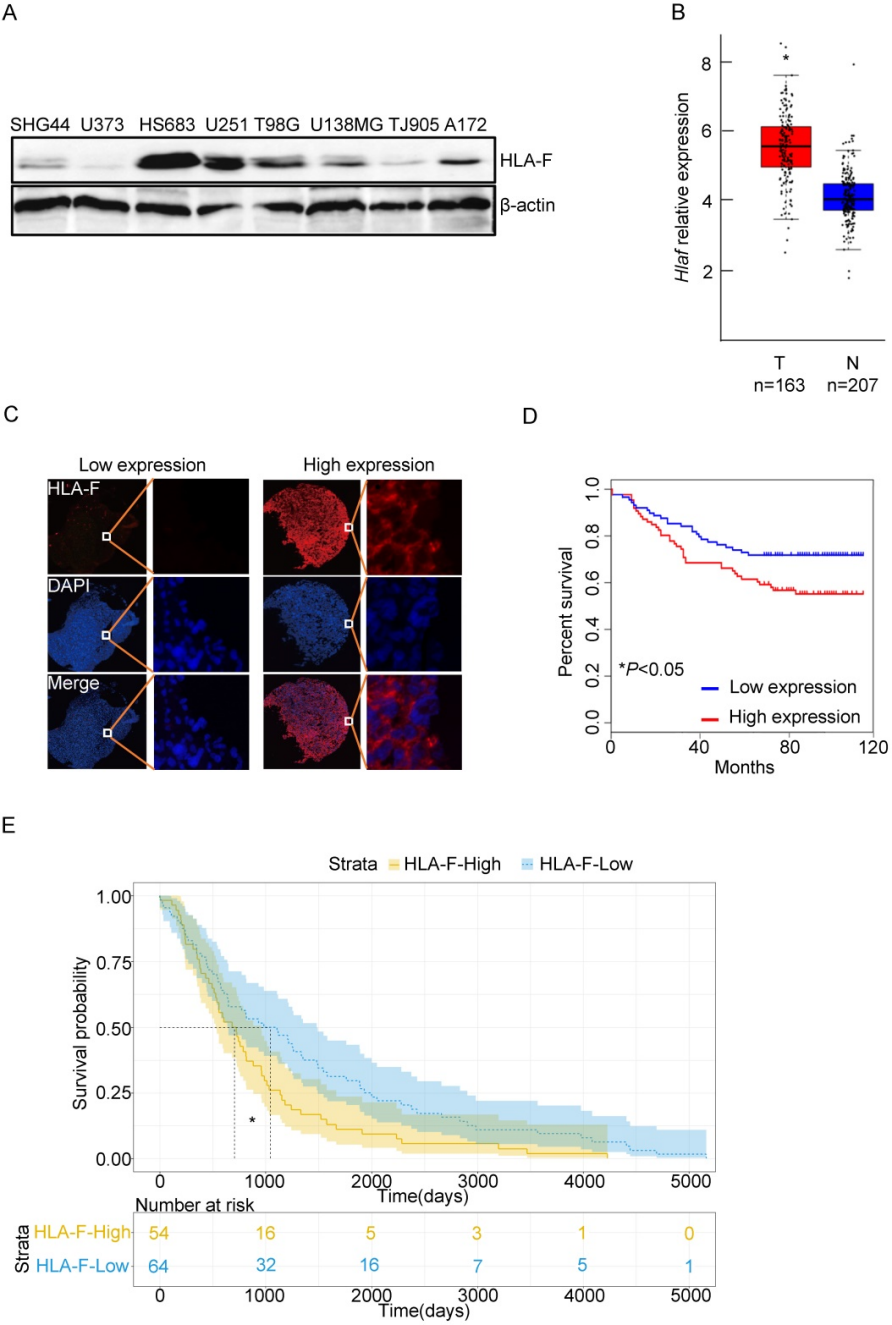
** The expression of HLA-F was closely associated with the outcome of GM patients. (A)** Western blot analysis. Proteins were extracted from the indicated GM cell lines and subjected to Western blot analysis. The membrane was sequentially probed with anti-HLA-F and anti-β-actin antibodies. The data represents one of two independent experiments with similar results. **(B)** The relative mRNA levels of *Hlaf* in GM tumor tissues (Red box, n = 163) and normal tissues (blue box, n = 207) were assessed using the GEPIA web tool. The relative expression level of *Hlaf* in each sample was shown as log2^FPKM^. *P < 0.05. **(C)** Immunofluorescence staining. Immunofluorescence for HLA-F (red) and DAPI (blue) in tumor sections (n = 180) was performed. The data shown represents a single experiment. **(D)** Kaplan-Meier plot indicating the overall survival of patients with GM categorized by HLA-F expression. The *P* value was determined by the log-rank test. *P < 0.05. **(E)** High HLA-F expression level was a risk factor for glioma patient survival, as determined by TCGA database analysis. *P < 0.05.

To determine the role of HLA-F in GM, we first examined the levels of HLA-F in a variety of GM cell lines, including HS683 and U251 cells, and found that HLA-F could be detected in GM cells (Figure 1A). We next explored whether HLA-F expression was increased in GM patients through the GEPIA web tool and found that *Hla-f* levels were significantly increased in individuals with GM (n = 163) compared with normal individuals (n = 207) (Figure 1B). Additionally, immunofluorescence staining revealed high expression of HLA-F in GM patients (Figure 1C). Consistent with a previous study 9, HLA-F expression was associated with GM patient survival (Figures 1D and 1E). Additionally, high expression of HLA-F could predict the progression and recurrence of GM (Table 2). As shown in the multivariate Cox regression analysis, the expression of HLA-F was an independent predictor of GM patient survival, together with age, stage and proliferating cell nuclear antigen (PCNA), an independent biomarker of overall survival 33 (Tables 3 and 4). In addition to GM, the constitutive expression of HLA-F was associated with the overall survival of individuals with acute myeloid leukemia (AML) and stomach adenocarcinoma (STAD, data not shown).

### HLA-F promoted cell proliferation

**Figure 2:**
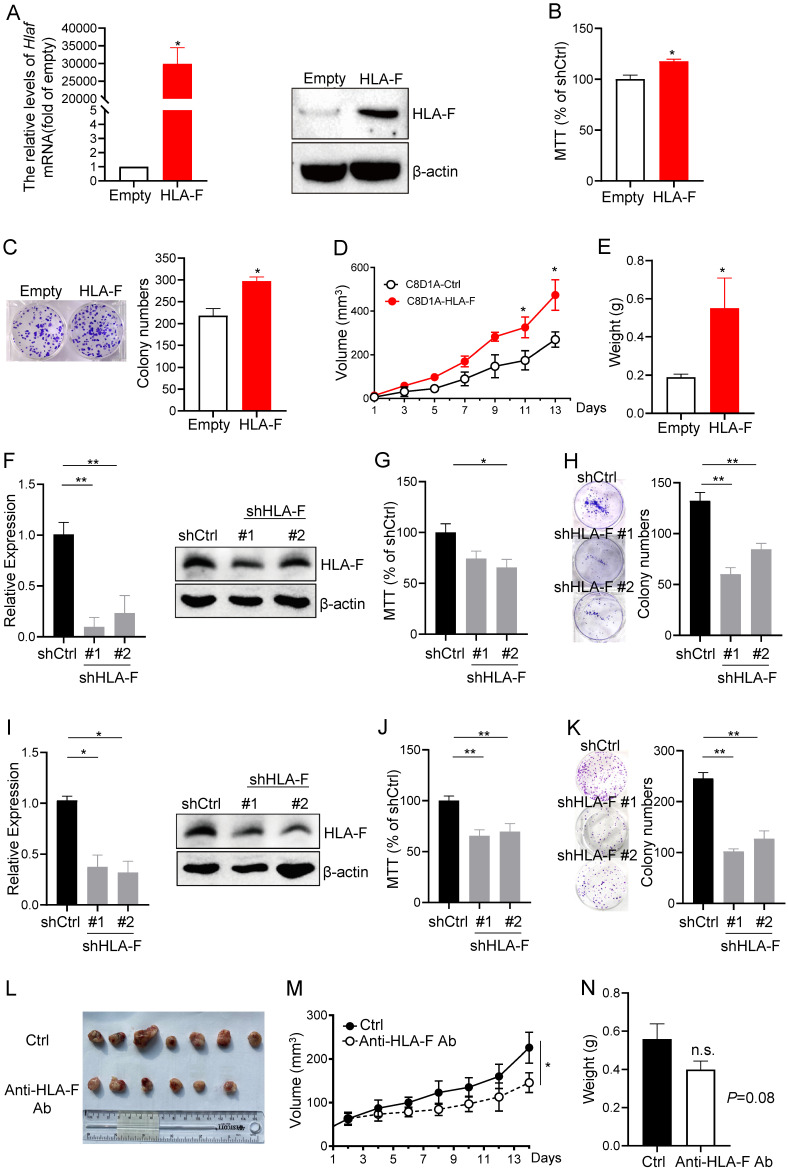
** HLA-F was involved in regulating cell proliferation. (A)** C8-D1A cells were infected with either empty lentivirus or HLA-F lentivirus and selected with puromycin for 14 days. Then, the relative expression of HLA-F mRNA (left) in C8-D1A cells infected with either empty lentivirus or HLA-F lentivirus was examined by real-time RT-PCR. Significance was calculated using Student's t-test. *P < 0.05. Western blot analysis (right). Proteins were extracted from either empty lentivirus or HLA-F lentivirus-infected C8-D1A cells and subjected to Western blot analysis. The membrane was sequentially probed with anti-HLA-F and anti-β-actin antibodies. The data demonstrates one of three independent experiments with similar results. **(B)** MTT assay. C8-D1A cells were infected with either control or HLA-F lentivirus and selected with puromycin for 14 days. Then, 1 × 10^4^ infected cells were plated into 96-well plates. After 24 h, cell viability was measured by the MTT assay. **(C)** Colony formation assay. Two hundred empty lentivirus- or HLA-F lentivirus-infected C8-D1A cells were plated into 6-well plates. After 10 days, visible colonies were fixed and stained with crystal violet, and the number of colonies was counted by ImageJ software. **(D)** C8-D1A cells (1 × 10^6^) were transplanted into *Rag2^-/-^* mice (n = 6 mice/group), and cell growth was monitored every other day. **(E)** The palpable mass was removed and weighed after 14 days. (B-E) The data demonstrate the mean ± SD of one of two independent experiments. Significance was calculated using Student's t-test. *P < 0.05. **(F)** HS683 cells were transfected with either control shRNA (shCtrl) or shRNAs against HLA-F (shHLA-F). After 24 h, the relative expression of HLA-F mRNA (left) in transfected HS683 cells was assessed by real-time RT-PCR. At the same time, protein (right) was extracted from transfected HS683 cells and subjected to Western blot analysis. The membrane was sequentially probed with anti-HLA-F and anti-β-actin antibodies. The data shows one of three independent experiments. **(G)** MTT assay. HS683 cells (1 × 10^4^) transfected with either shCtrl or shHLA-F were plated into 96-well plates. After 24 h, cell viability was measured by the MTT assay. **(H)** Colony formation assay. Two hundred transfected HS683 cells were plated into 6-well plates containing DMEM with 10% FBS in the presence of 2 μg/mL puromycin. 10 days later, colony number was counted by ImageJ software after crystal violet staining. (F-H) The bar graphs present the mean ± SD of one of three independent experiments performed in triplicate with similar results. One-way ANOVA followed by Dunnett's multiple comparisons test was utilized to calculate the *P* value. *P < 0.05, **P < 0.01. **(I)** U251 cells were transfected with either shCtrl or shHLA-F. After 24 h, the relative expression of HLA-F mRNA (left) in transfected U251 cells was assessed by real-time RT-PCR. At the same time, protein (right) was extracted from transfected U251 cells and subjected to Western blot analysis. The membrane was sequentially probed with anti-HLA-F and anti-β-actin antibodies. The picture shows one of three independent experiments. **(J)** MTT assay. U251 cells (1 × 10^4^) transfected with either shCtrl or shHLA-F were plated into 96-well plates. After 24 h, cell viability was measured by the MTT assay. **(K)** Colony formation assay. Two hundred transfected U251 cells were plated into 6-well plates containing DMEM with 10% FBS in the presence of 2 μg/mL puromycin. After 10 days, colony number was counted by ImageJ software after crystal violet staining. **(I-K)** The results are the mean ± SD of one of three independent experiments performed in triplicate with similar results. One-way ANOVA followed by Dunnett's multiple comparisons test was utilized to calculate the *P* value. *P < 0.05, **P < 0.01. **(L)** HLA-F-expressing C8-D1A cells (1 × 10^6^) were transplanted into *Rag2^-/-^* mice. When HLA-F-expressing C8-D1A cells formed a palpable mass, the mice were randomized to receive either the same volume control diluent (n = 7) or 100 µg/tumor anti-HLA-F mAb (n = 6) by intratumor injection. Representative images of palpable mass excised from *Rag2^-/-^* mice. (M) Growth curve of C8-D1A cells subjected to control treatmentor the anti-HLA-F monoclonal antibody treatment. *P < 0.05. **(N)** The palpable mass was removed and weighed at the end of the experiment. The results represent the mean ± SD of the weights. n.s., not significant. **(L-N)** The data shown represent one of two independent experiments with n = 6-7 mice/group. Significance was calculated using Student's t-test.

Given the above observations that aberrant expression of HLA-F may increase the proliferation index of GM cells, we overexpressed HLA-F in C8-D1A cells and performed MTT and colony formation assays. We revealed that the mRNA and protein levels of HLA-F were dramatically increased (Figure 2A). Interestingly, the viability and colony number of HLA-F-expressing C8-D1A cells were significantly elevated compared with those of empty vector-expressing C8-D1A cells (Figures 2B and 2C). Since HS683 and U251 cells highly expressing HLA-F barely formed tumors, to confirm whether HLA-F promotes cell proliferation in the mouse model, we transplanted HLA-F-expressing C8-D1A cells into *Rag2^-/-^* mice. As expected, HLA-F promoted C8-D1A cell growth in *Rag2^-/-^* mice (Figures 2D and 2E). To further validate the potential involvement of HLA-F in the proliferation of GM, we knocked down HLA-F and monitored the viability and colony formation of these cells. As shown in Figure 2F, shRNA against HLA-F decreased HLA-F expression in HS683 cells. As expected, silencing HLA-F significantly suppressed proliferation (Figures 2G and 2H). Similarly, knockdown of HLA-F also inhibited U251 cell growth (Figures 2I-2K). To further investigate whether HLA-F was involved in regulating proliferation *in vivo*,* Rag2^-/-^* mice transplanted with HLA-F-expressing C8-D1A cells were treated with either control diluent or anti-HLA-F antibody. The anti-HLA-F monoclonal antibody reduced the proliferation of HLA-F-expressing C8-D1A cells transplanted into *Rag2^-/-^* mice (Figures 2L-2N). These observations indicated that beyond its classic role in immunity, HLA-F could also be critical for controlling cell proliferation.

### HLA-F induced HK2 expression

**Figure 3:**
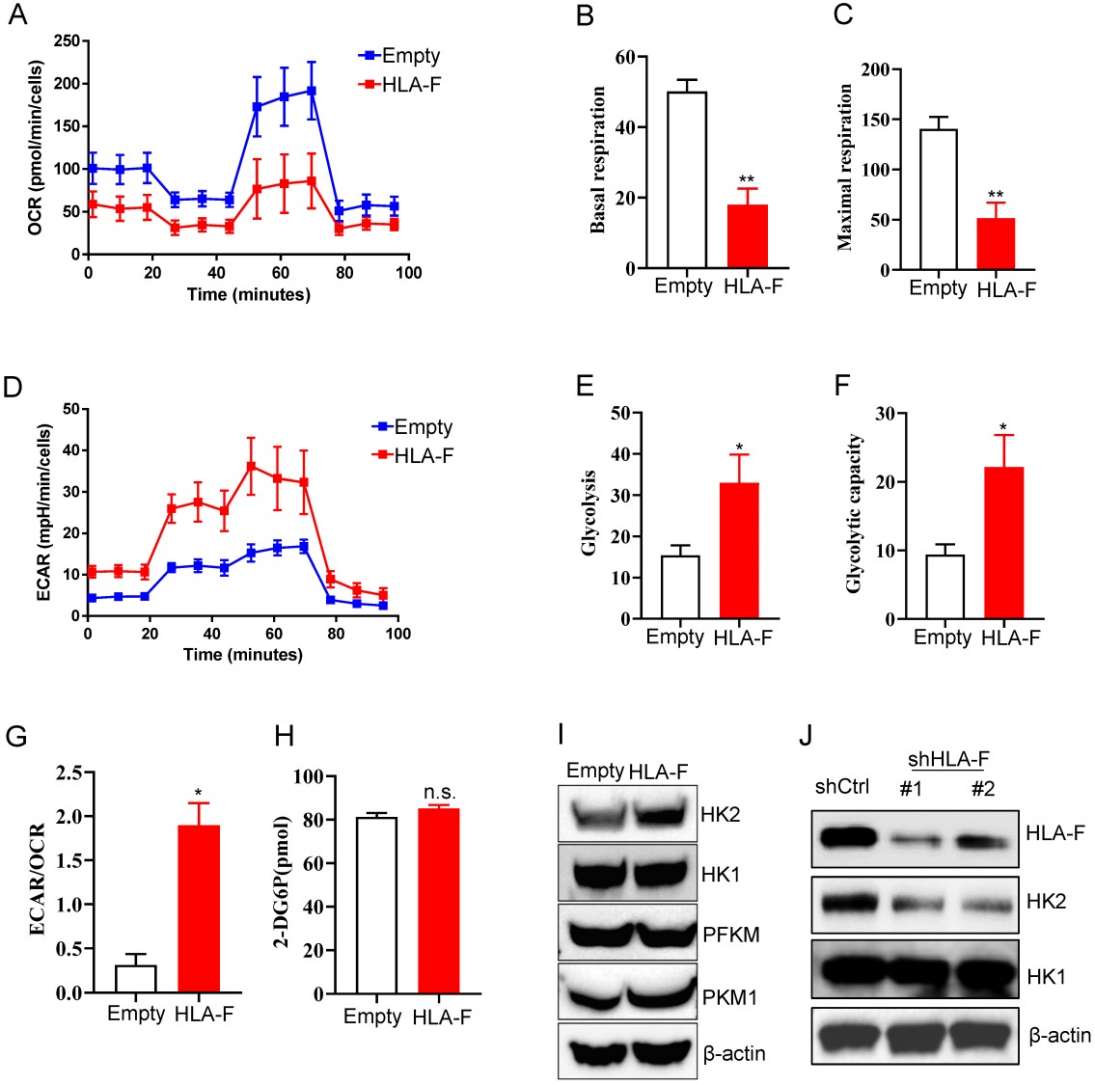
** Forced expression of HLA-F resulted in increased glycolysis. (A)** Empty vector-expressing C8-D1A cells and HLA-F-expressing C8-D1A cells were plated into wells, and the OCR was determined by extracellular flux analysis. A representative plot of OCR over time in cells treated with oligomycin (1 μM), FCCP (1 μM), and the electron transport inhibitors antimycin (100 nM) + rotenone (0.5 μM), as indicated. **(B)** Quantification of the basal respiration in Figure 3A. **(C)** Quantification of the maximal respiration of Figure 3A. **(A-C)** The data shown are representative of one of two independent experiments. **P < 0.01. **(D)** Empty vector-expressing C8-D1A cells and HLA-F-expressing C8-D1A cells were plated into wells, and ECAR was determined by extracellular flux analysis. A representative plot of ECAR over time in cells after the addition of glucose (100 mM), oligomycin (1 μM), and 2-DG (500 mM) as indicated. **(E)** Quantification of glycolysis in Figure 3D. **(F)** Quantification of the glycolytic capacity in Figure 3D. (D-F) The data shown are representative of one of two independent experiments. *P < 0.05. **(G)** Bar graph representing ratios of extracellular acidification rates (ECAR, indicator of aerobic glycolysis) to O_2_ consumption rates (OCR, indicator of OXPHOS) at baseline. The result represents the mean ± SD. The data shown is representative of one of two independent experiments. *P < 0.05. **(H)** The levels of glucose uptake in empty vector- or HLA-F-expressing C8-D1A cells were examined. The data shown is representative of one of two independent experiments. n.s., not significant. **(I)** Western blot analysis. Proteins were extracted from empty vector- and HLA-F-expressing C8-D1A cells and subjected to Western blot analysis. The membrane was sequentially probed with the indicated antibodies. The data shown are representative of one of two independent experiments. **(J)** Western blot analysis. Proteins were extracted from HS683 cells transfected with either control or shHLA-F and subjected to Western blot analysis. The membrane was sequentially probed with the indicated antibodies. The data shown are representative of one of two independent experiments.

To adapt to an environment deficient in oxygen, nutrients, and stimuli, cells are forced to reprogram their metabolism to serve their energy needs. Similar to the process of motility, the process of proliferation also poses high energy requirements. Normally differentiated cells primarily rely on mitochondrial oxidative phosphorylation (OXPHOS) to generate the energy needed for cellular processes 30. Therefore, we investigated whether metabolic changes occurred in response to the increase in HLA-F-mediated proliferation. To address this question, we measured the mitochondrial oxygen consumption rate (OCR) and the extracellular acidification rate (ECAR) in empty vector- and HLA-F-expressing C8-D1A cells. Unexpectedly, the OCR was decreased in HLA-F-infected C8-D1A cells compared to empty vector-infected C8-D1A cells (Figure 3A). Basal and maximal respiration was obviously reduced in HLA-F-expressing C8-D1A cells compared with empty vector-expressing C8-D1A cells (Figures 3B and 3C). Interestingly, we found that HLA-F-expressing C8-D1A cells displayed a higher basal ECAR than empty vector-expressing C8-D1A cells (Figures 3D and 3E). In addition, in contrast to empty vector-expressing C8-D1A cells, HLA-F-expressing C8-D1A cells had a higher glycolytic capacity (Figure 3F) and ECAR/OCR ratio (Figure 3G). These observations indicate that HLA-F-expressing C8-D1A cells could utilize glycolysis rather than mitochondrial OXPHOS.

To investigate the underlying molecular mechanism by which HLA-F regulates the high glycolytic phenotype, we first examined whether HLA-F-expressing C8-D1A cells had a much higher glucose uptake rate than their parental cells. As shown in Figure 3H, ectopic expression of HLA-F did not enhance glucose uptake. The finding that HLA-F increased glycolysis without affecting glucose uptake prompted us to ask whether HLA-F modulates the expression of enzymes involved in glucose metabolism. Therefore, we investigated whether HLA-F influences the expression of glycolytic enzymes and found that the protein level of HK2, a member of the HK family involved in catalyzing the first committed step in glucose metabolism 34, was increased in HLA-F-expressing C8-D1A cells (Figure 3I). However, the expression levels of HK1 and PFKM were almost identical in HLA-F-expressing C8-D1A cells compared with empty vector-expressing C8-D1A cells (Figure 3I). The levels of PKM1 were slightly increased in HLA-F-expressing C8-D1A cells compared with empty vector-expressing C8-D1A cells (Figure 3I). On the other hand, targeting HLA-F by shRNA suppressed HK2 protein expression in HS683 cells compared to HS683 cells transfected with control shRNA (Figure 3J). However, shRNA against HLA-F did not influence the expression of HK1 (Figure 3J). These results indicated that HLA-F could be involved in regulating cell proliferation through the induction of HK2 expression.

### HLA-F promoted proliferation by regulating HK2

**Figure 4:**
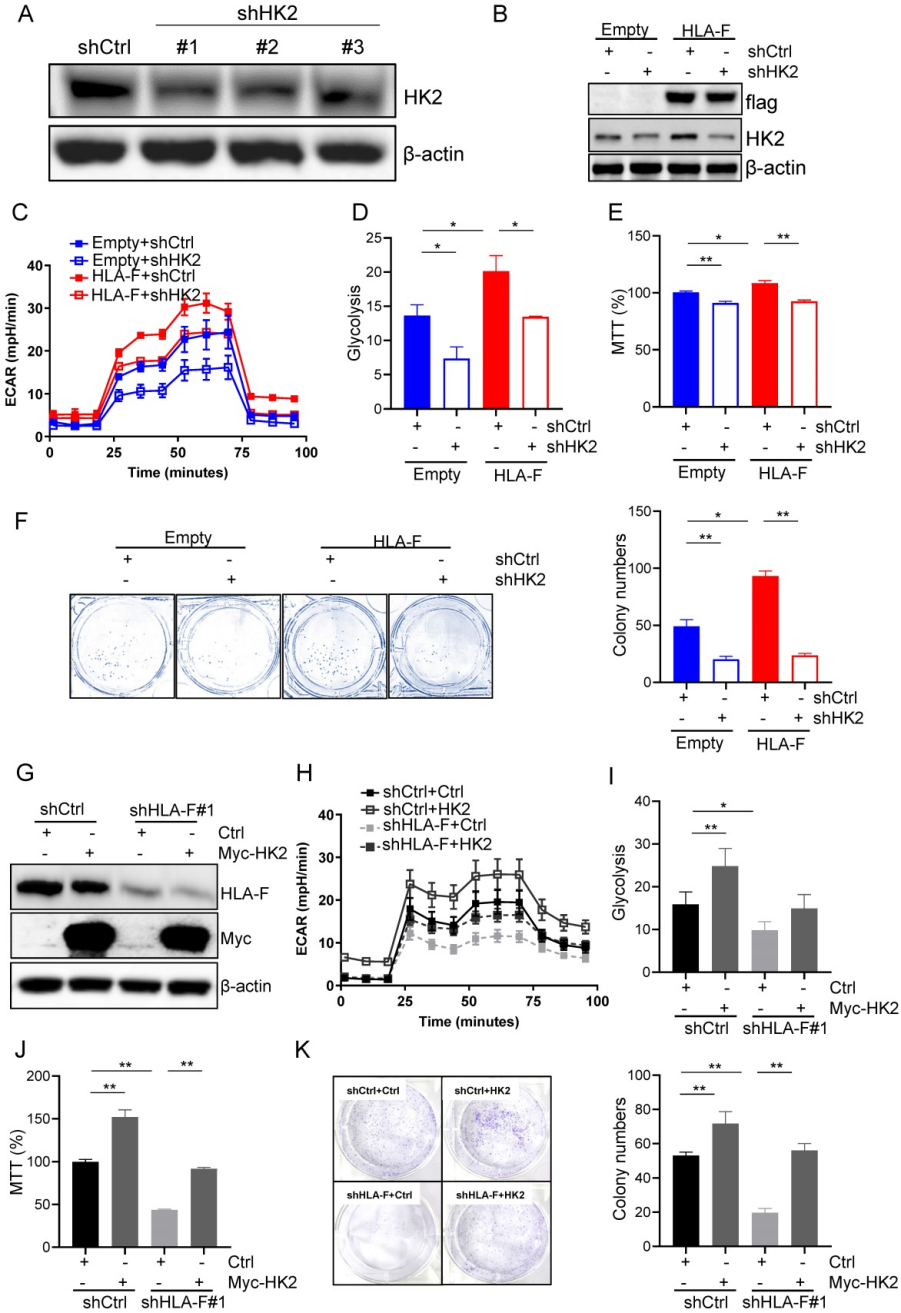
** HK2 was required for HLA-F-mediated proliferation and glycolysis. (A)** Western blot analysis. Proteins were extracted from C8-D1A cells transfected with either control or shHK2 and subjected to Western blot analysis. The membrane was sequentially probed with anti-HK2 and anti-β-actin antibodies. The data shown are representative of one of two independent experiments. **(B)** Western blot analysis. Proteins were extracted from empty vector- or HLA-F-expressing C8-D1A cells transfected with either shCtrl or shHK2 and subjected to Western blot analysis. The membrane was sequentially probed with anti-flag, anti-HK2 and anti-β-actin antibodies. The data represent one of two independent experiments. **(C)** Empty vector-expressing C8-D1A cells and HLA-F-expressing C8-D1A cells were transfected with either shCtrl or shHK2, and ECAR was determined by extracellular flux analysis. A representative plot of ECAR over time in these cells after the addition of glucose (100 mM), oligomycin (1 μM), and 2-DG (500 mM) as indicated. **(D)** Quantification of glycolysis in Figure 4C. **(E)** MTT assay. A total of 1 × 10^4^ empty vector-expressing C8-D1A cells and HLA-F-expressing C8-D1A cells transfected with either control or shHK2 were plated into 96-well plates. After 24 h, cell viability was measured by the MTT assay. **(F)** Colony formation assay. 200 empty vector- and HLA-F-expressing C8-D1A cells transfected with either control or shHK2 were plated into 6-well plates containing DMEM and 10% FBS in the presence of 2 μg/mL puromycin. 10 days later, the colonies were stained with crystal violet, and the number of colonies was counted by ImageJ software. (C-F) The bar graphs are the mean ± SD of one of two independent experiments performed in duplicate with similar results. One-way ANOVA followed by Tukey's multiple comparisons test was utilized to calculate the *P* value. *P < 0.05, **P < 0.01. **(G)** Western blot analysis. HS683 cells were treated with shRNA against HLA-F or nonspecific sequences (NS) followed by transfection with either empty vector or HK2 plasmid DNA for 24 h. Cell lysates were harvested and blotted for the indicated proteins. The data shown is representative of one of two independent experiments. **(H)** HS683 cells were treated with shRNA against HLA-F or non-specific sequences (NS) followed by transfection with either empty or HK2 plasmid DNA, and ECAR was determined by extracellular flux analysis. **(I)** Glycolysis of Figure 4H was quantified. **(J)** MTT assay. 1 × 10^4^ HS683 cells were treated with shRNA against HLA-F or non-specific sequences (NS) followed by transfection with either empty or HK2 plasmid DNA and planted into 96-wells. After 24 h, the viability of transfected cell was measured by MTT. **(K)** Colony formation assay. HS683 cells were treated with shRNA against HLA-F or nonspecific sequences (NS) followed by transfection with either empty vector or HK2 plasmid DNA. After 24 h later, the transfected cells were exposed to 2 μg/mL puromycin and 0.5 μg/mL G418 for 7 days. Two hundred selected cells were plated into 6-well plates containing DMEM and 10% FBS in the presence of 2 μg/mL puromycin and 0.5 μg/mL G418 for 7 days. Ten days later, the number of colonies was counted by ImageJ software after crystal violet staining. **(H-K)** The data shown are the mean ± SD of one of two independent experiments performed in duplicate with similar results. One-way ANOVA followed by Tukey's multiple comparisons test was utilized to calculate the *P* value. *P < 0.05, **P < 0.01.

To confirm that HLA-F promoted proliferation by modulating HK2 expression, we silenced HK2 with shRNA and performed Seahorse and proliferation assays. shRNA #1 and #2 against HK2 reduced HK2 expression in C8-D1A cells (Figure 4A). Herein, we utilized #1 shHK2 for subsequent experiments. Knockdown of HK2 effectively decreased HLA-F-mediated HK2 expression (Figure 4B). Additionally, the silencing of HK2 in HLA-F-expressing C8-D1A cells significantly decreased glycolysis compared to that in shCtrl-transfected cells (Figures 4C and 4D). Moreover, knocking down HK2 inhibited HLA-F-promoted cell proliferation (Figures 4E and 4F). On the other hand, ectopic expression of HK2 in HS683 cells transfected with shHLA-F increased glycolysis and the colony numbers compared with their parental cells (Figures 4G-4K). These observations demonstrated that HK2 could be critical for HLA-F-mediated cell proliferation.

### HLA-F was required for HK2 protein stabilization

**Figure 5:**
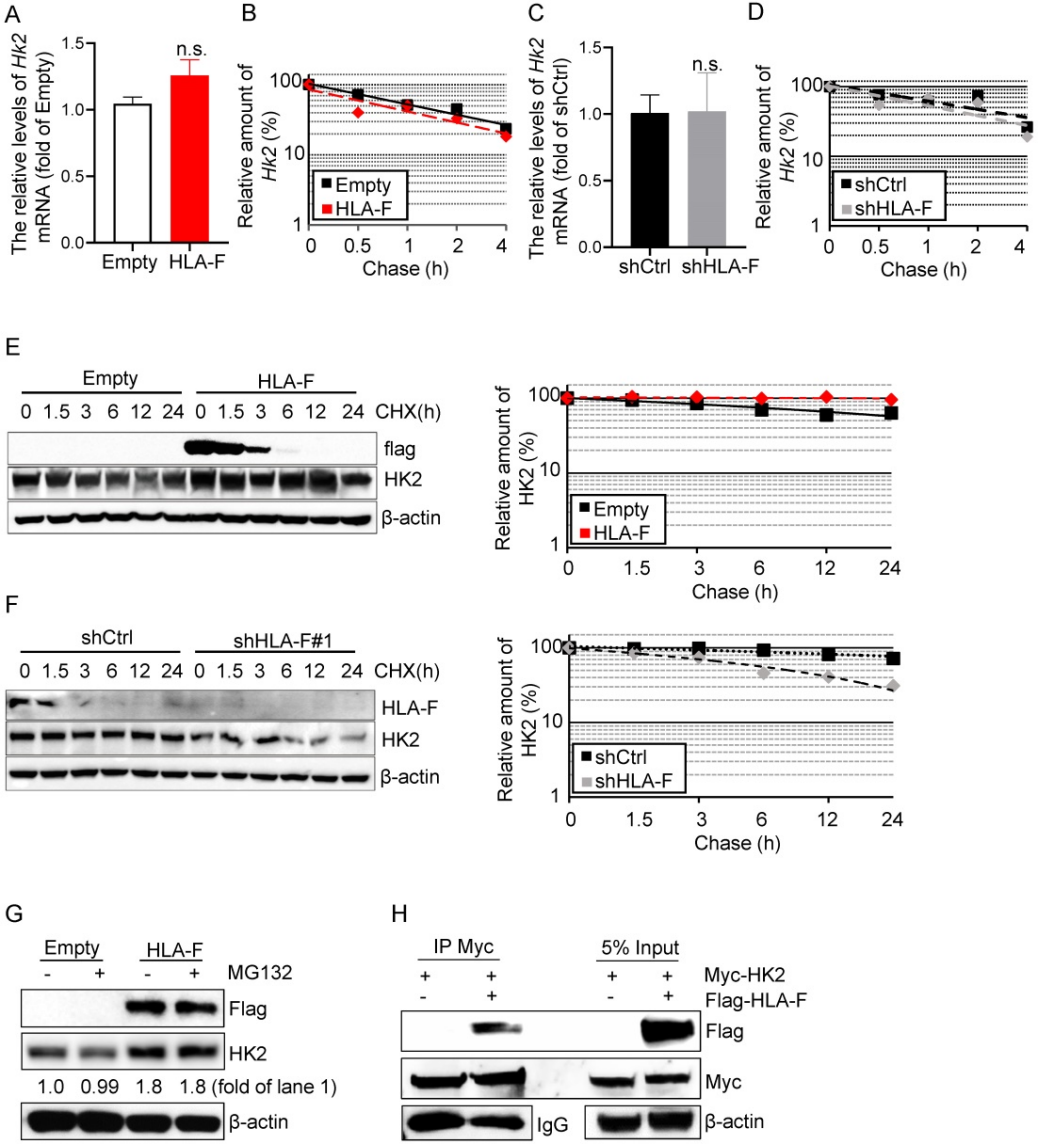
** HLA-F was involved in mediating HK2 protein stabilization. (A)** C8-D1A cells were infected with either empty lentivirus or HLA-F lentivirus and selected with puromycin for 14 days. The relative expression of *Hk2* in C8-D1A cells infected with either empty lentivirus or HLA-F lentivirus was examined by real-time RT-PCR. n.s., not significant. **(B)** Actinomycin D was added into the wells for the indicated timepoints to block RNA synthesis. *Hk2* mRNA was analyzed using real-time RT-PCR in C8-D1A cells infected with either empty lentivirus or HLA-F lentivirus. **(C)** The relative expression of *Hk2* in HS683 cells transfected with either control shRNA or shRNA against HLA-F was examined by real-time RT-PCR. n.s., not significant. **(D)** Actinomycin D was added for the indicated timepoints. *Hk2* mRNA was analyzed using real-time RT-PCR in HS683 cells transfected with either control shRNA or shRNA against HLA-F. (A-D) The data represent one of three independent experiments performed in triplicate with similar results. Significance was calculated using Student's t-test. **(E)** C8-D1A cells infected with either empty lentivirus or HLA-F lentivirus were treated with CHX at a final concentration of 100 μg/mL. At the indicated timepoints, protein was extracted, and the levels of flag, HK2, and β-actin were analyzed by Western blotting. The amount of HK2 in these cells was quantified by densitometry, normalized to the level of β-actin, and plotted. The data shown are representative of one of two independent experiments. **(F)** HS683 cells transfected with either shCtrl or shHLA-F were treated with CHX for the indicated timepoints. Protein was extracted, and the levels of HLA-F, HK2 and β-actin were analyzed by Western blotting. The amount of HK2 in these cells was quantified by densitometry, normalized to the level of β-actin, and plotted. The data shown are representative of one of two independent experiments. **(G)** Empty vector-expressing or HLA-F-expressing C8-D1A cells were treated with 5 μM MG-132 for 5 h before harvesting. The membrane was probed with the indicated antibodies. The data shown are representative of one of two independent experiments. **(H)** 293T cells were cotransfected with the indicated plasmid DNA. After 24 h, the cell lysates were isolated and immunoprecipitated with an anti-Myc antibody and analyzed by Western blotting using an anti-flag antibody.

HK2 is an attractive drug target against human cancers 16, 35, 36, which has prompted a number of investigations into the underlying molecular basis of HK2 regulation in cancers. HK2 is regulated at the mRNA and protein levels 37. To determine the mechanisms by which HLA-F controls HK2 expression, we first performed real-time RT-PCR and found that the mRNA levels of *Hk2* were almost identical in HLA-F-expressing C8-D1A cells compared to their parental cells (Figure 5A). Additionally, forced expression of HLA-F did not influence the stabilization of *Hk2* mRNA in C8-D1A cells (Figure 5B). Furthermore, knockout of HLA-F by shRNA did not affect the *Hk2* transcriptional levels or the stabilization of *Hk2* mRNA in HS683 cells (Figures 5C and 5D). Since HLA-F acts as a chaperone and stabilizes HLA class I molecules that have not yet bound peptides, we hypothesized that HLA-F could be involved in regulating the stabilization of the HK2 protein. We next examined the half-life of HK2 in the presence or absence of HLA-F. As expected, HK2 degradation was slower in HLA-F-expressing C8-D1A cells than in their parental cells (Figure 5E). In contrast, silencing HLA-F in HS683 cells induced the degradation of HK2 compared to that in HS683 cells transiently transfected with shCtrl (Figure 5F). To examine whether HLA-F affects HK2 stability through ubiquitination degradation, we treated empty vector-expressing or HLA-F-expressing C8-D1A cells with MG-132 and found that HK2 expression did not change in the presence of MG-132 (Figure 5G). To further explore the relationship between HLA-F and HK2, we also performed co-IP and found that HLA-F interacted with HK2 (Figure 5H), indicating that HLA-F was critical for HK2 stabilization.

## Discussion

HLA-F, a nonclassical HLA-Ib molecule, has been indicated to be critical for human pregnancy, infection and autoimmunity 8, 11, 12. A large number of studies found that HLA-F is aberrantly expressed in several types of cancers 9, 13, 14 and is associated with overall survival in cancer patients 9, 13. The underlying mechanism of HLA-F activity remains obscure, and only recently has it become clear that HLA-F is an important immune regulator 38. Recent data indicated that HLA-F interacts with both activating and inhibitory receptors expressed on immune cells, which could present a diverse panel of peptides to T cells 39-41. It has also been shown that HLA-F can present peptides to T cells and can regulate immunity via interactions with distinct NK cell receptors, which depends on the molecular conformation of peptide-bound HLA-F or HLA-F open conformers 41, highlighting the function of HLA-F as an important immunoregulatory molecule. However, in addition to acting as an immunoregulatory molecule, the roles of HLA-F in tumor development are unclear.

Consistent with a previous study 9, we also found that HLA-F was constitutively expressed in GM (Figure 1). High expression of HLA-F predicted the progression and recurrence of GM (Table 2). Interestingly, aberrant expression of HLA-F resulted in a high cell proliferation index both *in vitro* and *in vivo* (Table 2, Figure 2). It has been generally accepted that proliferating cells utilize glycolysis to assist their growth 36. A shift to glycolysis resulting from aberrant regulation of glycolytic enzymes was observed in several types of cancers 19, 42-44. Notably, in this study, a shift to glycolysis occurred in HLA-F-expressing C8-D1A cells along with an increase in HK2 expression (Figure 3). Silencing of HK2 suppressed HLA-F-mediated cell proliferation in parallel with a decrease in glycolysis (Figure 4). On the other hand, ectopic expression of HK2 in HS683 cells transfected with shHLA-F increased the colony number (Figure 4). These observations suggested that HLA-F-induced cell proliferation could depend on HK2-mediated glycolysis. In addition, elevated glycolysis with similar glucose uptake levels suggested pentose phosphate pathway (PPP) activity, which is very relevant for nucleotide biosynthesis, and the generation of reducing power (NADPH) 45 could be decreased in HLA-F-expressing GM cells. As expected, glucose-6-phosphate dehydrogenase (G6PDH) activity was decreased in HLA-F-expressing C8-D1A cells (Supplementary Figure 1), indicating that HLA-F-expressing GM cells might be sensitive to reactive oxygen species or ribose shortages, which should be investigated in further studies. Moreover, HLA-F expressed in some tissues and immune cells, and may be involved in altering the activation threshold of immune effector cells and mediating the activation of immune cells, such as activated regulator T cells 40, 46. If blocking HLA-F by antibody in the mouse model could influence the functions of immune cells are unclear, which need to be clarified in further experiments.

Unlike HK1, HK2 is highly expressed in a variety of tumors, including GBM 42, and is a key mediator of glycolysis in GBM 19. Silencing of HK2, but not HK1, by siRNA inhibited tumor growth in a xenograft model of GBM 19. Similarly, knocking down HK2 by shRNA suppressed the proliferation of HLA-F-expressing C8-D1A cells (Figure 4). Constitutive expression of HK2 could protect cancer cells from apoptosis and provide a survival and proliferating advantage *in vivo* 42. Additionally, HK2 plays an important role in tumor initiation and maintenance 32. The metabolic switch is one of the characteristic hallmarks of cancer cells and contributes to tumor development 47. All of these observations suggest that beyond its classical roles in immunity, HLA-F might function as an “oncogenic driver” in the progression of GBM by regulating HK2-dependent glycolysis, at least partially, although we cannot rule out the roles of HLA-F in allowing cancer cells to escape immune system surveillance *in vivo*.

Our study showed that HK2 protein expression was elevated in the presence of HLA-F, while HK1 expression did not change significantly. The underlying molecular basis for the selective induction of HK2 expression in HLA-F-expressing C8-D1A cells may be related to the fact that HLA-F interacted with HK2 and enhanced the stabilization of the HK2 protein (Figure 5), although the mechanisms by which HLA-F is involved in regulating HK2 stabilization are unclear. Moreover, HK2 has been shown to translocate into the outer mitochondrial membrane and interact with voltage-dependent anion channels (VDACs) 21, 22. Knocking down HK2 dramatically increased the expression of OXPHOS-associated proteins involved in the electron transport chain, as well as the key transcription factors involved in mitochondrial function and biogenesis 19. All of these data further elucidate the role of HLA-F in suppressing OXPHOS (Figure 3).

In summary, our results demonstrated that HLA-F promoted cell proliferation by regulating HK2-dependent glycolysis. Additionally, our also indicated that HLA-F could be a potential therapeutic target for the treatment of GBM.

## Supplementary Material

Supplementary figure S1.

## Figures and Tables

**Table 1:** Real time RT-PCR primers

Gene	Direction	Primer
*Hk2*	Forward	5'-AGAAATGGAGCGAGGTCTGA-3'
	Reverse	5'-TTGTTCCTCCAAGGTCCAAG-3'
* Hlaf*	Forward	5'-AATGGGAAGGAGACGCTACA-3'
	Reverse	5'-CACAGCTCCAAGGACAACAA-3'
*18S*	Forward	5'-AAACGGCTACCACATCCAAG-3'
	Reverse	5'-CCTCCAATGGATCCTCGTTA-3'
*Actb*	Forward	5'-GCTACAGCTTCACCACCACA-3'
	Reverse	5'-TCTCCAGGGAGGAAGAGGAT-3'
*mHk2*	Forward	5'-GAAGATGATCAGCGGGATGT-3'
	Reverse	5'-GCCAGTGGTAAGGAGCTCTG-3'

**Table 2:** Relationship between the level of HLA-F and clinicopathological features of GM patients

Variables	HLA-F (n = 180 cases)				
	Total	High (%)	Low (%)	N/A	*P*
All patients	180	89 (100)	90 (100)	1 (100)	
**Sex**					0.205
male	112	50	61	1	
female	68	39	29	0	
**Age (years)**					0.523
<60	151	72	78	1	
≥60	29	17	12	0	
**Stage**					<0.001
I+II	105	39	65	0	
III+IV	75	50	25	0	
**Recurrence**					<0.001
yes	96	62	34	0	
no	84	27	56	0	
**PCNA**					0.031
N/A	85	40	45	0	
-/+	29	9	20	0	
++	60	34	25	1	
+++	6	6	0	0	
**GFAP**					0.024
N/A	14	6	8	0	
-/+	74	36	38	0	
++	80	41	39	0	
+++	12	6	5	1	
**S-100**					0.074
N/A	21	12	9	0	
-/+	86	41	45	0	
++	56	30	26	0	
+++	17	6	10	1	
**CK**					0.401
N/A	111	50	60	1	
-/+	59	31	28	0	
++	8	7	1	0	
+++	2	1	1	0	
**Vimentin**					0.979
N/A	122	60	61	1	
-/+	29	16	13	0	
++	25	11	14	0	
+++	4	2	2	0	

**Table 3:** Univariate Cox regression analysis of HLA-F expression and clinicopathological variables predicting the 5-year survival of GM patients

Variables	(n = 180 cases)	
	HR (95% CI)	*P*
Sex (male vs. female)	0.669 (0.384-1.164)	0.155
Age (<60 years vs. ≥60 years)	2.454 (1.382-4.359)	0.002
Stage (I+II vs. III+IV)	14.812 (6.997-31.360)	<0.001
PCNA	0.691 (0.508-0.941)	0.019
S-100	0.790 (0.577-1.080)	0.139
CK	0.845 (0.557-1.282)	0.429
Vimentin	1.297 (0.990-1.699)	0.059
Recurrence (yes vs. no)	0.011 (0.001-0.093)	<0.001
HLA-F (high vs. low)	1.752 (1.045-2.940)	0.034

HR: hazard ratio; CI: confidence interval; P values are from log-rank test.

**Table 4:** Univariate Cox regression analysis for recurrence-free survival of GM patients

Variables	(n= 180 cases)	
	HR (95% CI)	*P*
Sex (male vs. female)	0.736 (0.480-1.127)	0.158
Age (<60 years vs. ≥60 years)	1.864 (1.165-2.985)	0.009
Stage (I+II vs. III+IV)	6.871 (4.421-10.680)	<0.001
PCNA	0.658 (0.513-0.844)	0.001
S-100	0.730 (0.576-0.924)	0.009
CK	0.973 (0.726-1.306)	0.858
Vimentin	1.374 (1.118-1.687)	0.002
HLA-F (high vs. low)	2.297 (1.518-3.475)	<0.001

HR: hazard ratio; CI: confidence interval; P values are from log-rank test.

## Abbreviations

GM: glioma
GBM: glioblastoma
CD95L: CD95 ligand
HLA-I: HLA class I
HLA-Ib: nonclassical HLA class I molecule
KIRs: killer cell immunoglobulin-like receptors
HKs: hexokinases
mAb: monoclonal antibody
IPTG: isopropyl-1-thio-β-D-galactopyranoside
IDA: iminodiacetic acid
HAT: hypoxanthine-aminopterin-thymidine
HT: hypoxanthine-thymidine
ELISA: enzyme-linked immunosorbent assay
HRP: horseradish peroxidase
SPF: specific pathogen-free
MTT: 3‐(4,5‐dimethylthiazol‐2‐yl)‐2,5‐diphenyltetrazolium bromide
CHX: cycloheximide
SDS: sodium dodecyl sulfate
ECAR: extracellular acidification rate
OCR: oxygen consumption rate
FCCP: Carbonyl cyanide 4-(trifluoromethoxy)phenylhydrazone
OS: overall survival
LGG: low-grade glioma
TCGA: The Cancer Genome Atlas
PCNA: proliferating cell nuclear antigen
OXPHOS: oxidative phosphorylation
PPP: pentose phosphate pathway
G6PDH: glucose-6-phosphate dehydrogenase
