# Supplementary material for: Targeting HLA-F suppresses the proliferation of glioma cells via a reduction in hexokinase 2-dependent glycolysis
Source: Int J Biol Sci. 2021 Mar 25;17(5):1263–76. doi: 10.7150/ijbs.56357 (PMC8040476; doi:10.7150/ijbs.56357)
Supplement: Supplementary file 1 — Supplementary figure S1. [file ijbsv17p1263s1.pdf]

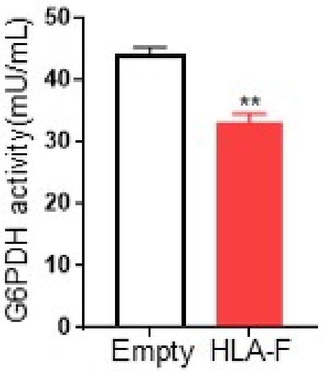

Supplementary Figure 1. G6PDH activity was lower in HLA-F expressing C8-D1A cells.

Protein was extracted from either empty expressing or HLA-F expressing C8-D1A cells and subjected to measure the G6PDH activity. The data shown are one of three independent experiments with similar results. **, $P < 0.01$.
